# Induction of Semaphorin 3A by Resveratrol and Pinostilbene via Activation of the AHR-NRF2 Axis in Human Keratinocytes

**DOI:** 10.3390/antiox13060732

**Published:** 2024-06-17

**Authors:** Gaku Tsuji, Ayako Yumine, Koji Kawamura, Masaki Takemura, Takeshi Nakahara

**Affiliations:** 1 Research and Clinical Center for Yusho and Dioxin, Kyushu University Hospital, Fukuoka 812-8582, Japan; yumine.ayako.977@m.kyushu-u.ac.jp (A.Y.); nakahara.takeshi.930@m.kyushu-u.ac.jp (T.N.); 2 Department of Dermatology, Graduate School of Medical Sciences, Kyushu University, Fukuoka 812-8582, Japan; kawamura.koji.570@s.kyushu-u.ac.jp (K.K.); takemura.masaki.618@m.kyushu-u.ac.jp (M.T.)

**Keywords:** atopic dermatitis, semaphorin 3A, keratinocyte, pruritus

## Abstract

Semaphorin 3A (SEMA3A), a nerve-repellent factor produced by keratinocytes, has an inhibitory effect on nerve extension to the epidermis. Epidermal innervation is involved in pruritus in inflammatory skin diseases such as atopic dermatitis (AD) and dry skin. We previously reported that tapinarof, a stilbene molecule, upregulates SEMA3A in human keratinocytes. We also showed that this mechanism is mediated via the aryl hydrocarbon receptor (AHR), a ligand-activated transcription factor, and the nuclear factor erythroid 2-related factor 2 (NRF2) axis. Since some stilbenes activate AHR and NRF2, we attempted to identify other stilbenes that upregulate SEMA3A. We analyzed normal human epidermal keratinocytes (NHEKs) treated with 11 types of stilbenes and examined SEMA3A expression. We found that resveratrol and pinostilbene, antioxidant polyphenols, upregulated SEMA3A and increased nuclear AHR and NRF2 expression. In addition, AHR knockdown by small interfering RNA (siRNA) transfection abolished the NRF2 nuclear expression. Furthermore, AHR and NRF2 knockdown by siRNA transfection abrogated resveratrol- and pinostilbene-induced SEMA3A upregulation. Finally, we confirmed that resveratrol and pinostilbene increased SEMA3A promoter activity through NRF2 binding using ChIP-qPCR analysis. These results suggest that resveratrol and pinostilbene upregulate SEMA3A via the AHR–NRF2 axis in human keratinocytes.

## 1. Introduction

Pruritus is a serious symptom that reduces quality of life in patients with inflammatory skin diseases such as atopic dermatitis (AD) [1]. AD develops as a chronic recurrent eczematous lesion with severe pruritus. AD affects approximately 20% of children and 5% of adults [2]. Disturbance of sleep due to pruritus contributes to anxiety, depression, and suicidal ideation in AD patients [3]. In addition, the intensity of pruritus correlates with the severity of AD [4]. Furthermore, scratch damage to keratinocytes induced by pruritus exacerbates AD by producing chemokines such as CCL20 and CXCL8, which recruit T cells and neutrophils to the epidermis [5]. The pathogenesis of AD involves multiple factors, including the following three main ones: skin barrier dysfunction, type II inflammation, and pruritus [6,7]. These are induced by type II immune responses centered on IL-4 and IL-13 [7]. Recently, it has been elucidated that IL-4 and IL-13 stimulate their receptors on sensory neurons, which evokes itch. Selective inhibitors of IL-4 and IL-13 such as biologics and JAK inhibitors have also been shown to reduce pruritus in AD patients [8]. However, these treatments may not sufficiently alleviate pruritus, and more effective treatments for pruritus in AD are still desired [9].

The mechanism of pruritus in AD has been shown to include increased peripheral nerve density in AD lesions and the presence of nerve fibers reaching the upper region of the epidermis [10]. Increased epidermal innervation has been observed in AD lesions [10,11], which is related to the neurosensitive state of AD, such as alloknesis (a hypersensitive state in which itching occurs at the slightest provocation) and hyperknesis (a state in which itching does not stop even after scratching) [12]. Epidermal innervation is reportedly mediated by the balance between nerve growth factor (NGF), which induces nerve growth, and semaphorin 3A (SEMA3A), which inhibits nerve growth [13]. It has been reported that SEMA3A expression is decreased in the epidermis of AD lesions, leading to epidermal innervation [13]. It has also been reported that increased epidermal nerve fiber density correlates with decreased Sema3A expression levels in a murine model of dry skin [14]. In addition, SEMA3A reportedly exerts anti-inflammatory effects on immune cells. Specifically, SEMA3A inhibits the migration of dendritic cells and macrophages, while also suppressing T-cell activation by blocking the MAP kinase signaling pathway [15]. Thus, the induction of SEMA3A in keratinocytes is expected to improve pruritus and ameliorate AD by inhibiting epidermal innervation and immune cell activation.

Recent studies have shown that sensory nerves produce factors that have significant effects on the immune system, such as substance P, calcitonin gene-related peptide, vasoactive intestinal peptide, and neuromedin U [16]. These factors induce increased production of type 2 cytokines by group 2 innate lymphocytes and Th2 cells, synergistically enhancing type 2 immune responses. They also induce angiogenesis and vascular endothelial adhesion molecule expression and promote inflammatory cell migration to the AD lesion [16,17]. Thus, epidermal innervation may be deeply involved in the formation of type II immune responses, not only by generating pruritus but also by acting on both innate and acquired immune cells.

Although few reports on the treatment of AD with SEMA3A in humans have been published, one report described that the topical application of SEMA3A protein improved pruritus and disease activity in an AD mouse model [18]. It has also been reported that baicalein, a component of Chinese herbal medicines, increases SEMA3A expression in human keratinocytes [19]. However, to the best of our knowledge, no reports on topical agents that increase SEMA3A expression have been published, and the mechanism by which SEMA3A is regulated remains unclear. Moreover, no receptors that act as sensors to increase SEMA3A expression have been identified.

We previously reported that tapinarof, a topical agent under development for the treatment of AD, increases SEMA3A expression in human keratinocytes [20]. Tapinarof is classified as a therapeutic aryl hydrocarbon receptor (AHR)-modulating agent (TAMA) [20]. AHR, a chemical sensor that transduces extrinsic and intrinsic signals into cellular responses, is highly expressed in the epidermis and is involved in keratinocyte differentiation and proliferation, inflammatory cytokine production, and immune regulation of Th17/22 cells and regulatory T cells [21]. The binding of endogenous and exogenous ligands to AHR results in translocation from the cytoplasm to the nucleus. Activated AHR functions as a transcription factor, causing the upregulation of drug-metabolizing enzymes such as CYP1A1 [22]. AHR also regulates the activation of nuclear factor erythroid 2-related factor 2 (NRF2), which induces the expression of cytoprotective genes encoding detoxification and antioxidant enzymes such as NAD(P)H:quinone oxidoreductase 1 (NQO1) [23,24]. Activated NRF2 translocates to the nucleus and binds to antioxidant-responsive element-like loci (AREL) in the promoter region of the target gene, which induces the expression of that gene [25]. The AHR-NRF2 axis is considered an important signaling pathway in the pharmacological effects of TAMA [23,26], and we demonstrated that the upregulation of SEMA3A induced by tapinarof is dependent on this axis [20].

Tapinarof is a natural stilbene compound [27,28], and some stilbenes have been reported to activate AHR and NRF2 [29,30]. Thus, there may be stilbenes that potentially produce as much or more SEMA3A than tapinarof. To test this, we treated normal human epidermal keratinocytes (NHEKs) with 11 different stilbenes and examined the resulting SEMA3A expression.

## 2. Materials and Methods

### 2.1. Chemicals

Resveratrol, pinostilbene, rhapontigenin, isorhapontigenin, pterostilbene, and gnetol were purchased from TCI Chemicals (Tokyo, Japan). Pinosylvin and palovarotene were from Cayman Chemical (Ann Arbor, MI, USA), 3,4,5,4′-tetramethoxystilbene and 2,4,3′,5′-tetramethoxystilbene were from Sigma-Aldrich (St. Louis, MI, USA), and tapinarof was from MedChemExpress (Monmouth Junction, NJ, USA).

### 2.2. Cell Culture

NHEKs were purchased from Lonza (Basel, Switzerland) and maintained in KGM gold keratinocyte growth medium supplemented with a SingleQuots kit (Lonza, Basel, Switzerland). NHEKs were used in the experiments after three to five passages.

### 2.3. Cell Viability Test

The effects of stilbenes on cell viability were measured using a Cell Counting Kit-8 (Dojindo, Tokyo, Japan) containing water-soluble tetrazolium salt (WST). NHEKs were seeded in a 96-well plate and treated with the indicated stilbenes for 24 h. WST solution was then added to the cells. The absorbance of each sample was measured using a microplate reader (iMark microplate reader; Bio-Rad Laboratories, Danvers, MA, USA) with a filter at 450 nm. The results are presented as the absorbance relative to that of untreated NHEKs. The results are shown in Supplementary Figure S1.

### 2.4. Quantitative Real-Time PCR (qPCR) Analysis

Total cellular RNA was prepared using an RNeasy Mini Kit (Qiagen, Hilden, Germany) and reverse-transcribed using a PrimeScript RT Reagent Kit (Takara Bio, Otsu, Japan). Real-time quantitative PCR was performed using TB Green Premix Ex Taq (Takara Bio) or TaqMan Fast Advanced Master Mix (Thermo Fisher Scientific, Waltham, MA, USA). Primers and probes are listed in Table S1. Relative mRNA expression was normalized by that of *YWHAZ*.

### 2.5. Small Interfering RNA Transfection

Small interfering RNA (siRNA) targeting NRF2 (s9492) or AHR (s1200) and scrambled RNA (Silence Negative Control No. 1) were purchased from Thermo Fisher Scientific. NHEKs were transfected with 5 nM siRNA using Lipofectamine RNAi Max (Thermo Fisher Scientific) in accordance with the manufacturer’s instructions.

### 2.6. Western Blot Analysis

Cells were washed with PBS, and cellular proteins were lysed with RIPA sample buffer containing 140 mM NaCl, 50 mM Tris-HCl, 1% NP-40, 0.5% sodium deoxycholate, 0.1% sodium dodecyl sulfate, 1 mM EDTA, and 1 mM NaF. A NE-PER nuclear and cytoplasmic extraction reagent kit (Thermo Fisher Scientific) was used to separate cytosolic and nuclear proteins. Equal amounts of protein were subjected to PAGE using 4–12% gels and transferred to PVDF membranes (Merck Millipore, Burlington, MA, USA). The membranes were then immunoblotted with primary antibodies. The antibodies used were SEMA3A (#23393; Abcam, Cambridge, UK), NRF2 [EP1808Y] (Abcam), histone deacetylase (HDAC) 1 (#5356, Cell Signaling Technology, Danvers, MA, USA), AHR [D5S6H] (Cell Signaling Technology), actin beta (ACTB) [8H10D10] (Cell Signaling Technology), anti-rabbit IgG HRP-conjugated (#7074; Cell Signaling Technology), and anti-mouse IgG HRP-conjugated (#7076; Cell Signaling Technology). Secondary antibodies were detected using Super Signal West Pico Plus Chemiluminescent Substrate (Thermo Fisher Scientific). Densitometric analysis of protein band was performed using Image Lab 5.2 (Bio-Rad Laboratories, Danvers, MA, USA).

### 2.7. Luciferase Assay on SEMA3A Promoter

Construction of the SEMA3A luciferase vector and mutant vectors was performed as described previously [20]. Briefly, a PCR fragment corresponding to −209 bp to +1 bp of the SEMA3A gene was cloned into a pGL4.14 luciferase vector (Promega, Madison, WI, USA). Point mutations were introduced by PCR mutagenesis using the Infusion Cloning Kit (Takara Bio).

A total of 2 × 10^4^ cells were seeded onto 96-well plates and cultured overnight. The next day, the cells were transiently transfected with 0.1 μg of plasmid DNA using X-tremeGENE HP DNA Transfection Reagent (Roche Applied Science, Penzberg, Germany). To calculate the transfection efficiency, all cells were co-transfected with the pSV-β-galactosidase control vector (Promega). Twenty-four hours after transfection, the culture medium was changed to the experimental conditions described in each figure legend. Cells were reacted with ONE-Glo luciferase assay buffer (Promega), and luciferase activity was measured using an Infinite 200 PRO microplate reader (Tecan Group Ltd., Männedorf, Switzerland). For the measurement of β-galactosidase activity, cells were lysed with reporter lysis buffer and reacted with 4 mM chlorophenol red β-d-galactopyranoside for more than 6 h. Absorbance at 570 nm was used to normalize the luciferase activity. Fold induction is expressed as the ratio of induction relative to that for mock vector-transfected cells.

### 2.8. ChIP-qPCR Assay

ChIP assays were performed with a SimpleChIP Plus Enzymatic Chromatin IP Kit (Cell Signaling Technology). NHEKs were fixed with 1% paraformaldehyde for 10 min at room temperature. Chromatin was sheared by the combination of enzymatic digestion with micrococcal nuclease, included in the kit, and sonication with BIORUPTOR II (Sonicbio Co., Ltd., Kanagawa, Japan). A total of 5 µg of digested chromatin was reacted with 0.4 µg of anti-NRF2 antibody or isotype control IgG overnight at 4 °C with rotation, and the samples were precipitated with protein A agarose. DNA was purified from input and precipitated samples and subjected to qPCR. The primers used are listed in Supplementary Table S1.

### 2.9. Statistical Analysis

Data are presented as mean ± standard deviation of three independent experiments. A two-tailed, paired *t*-test was used for statistical analysis between two groups. Dunnett’s multiple comparison test (nonparametric Steel test) or Tukey’s multiple comparison test was performed to determine the statistical significance of differences among three or more groups. A *p*-value of less than 0.05 was considered significant.

## 3. Results

### 3.1. Resveratrol and Pinostilbene Upregulated SEMA3A in NHEKs

**Figure 1:**
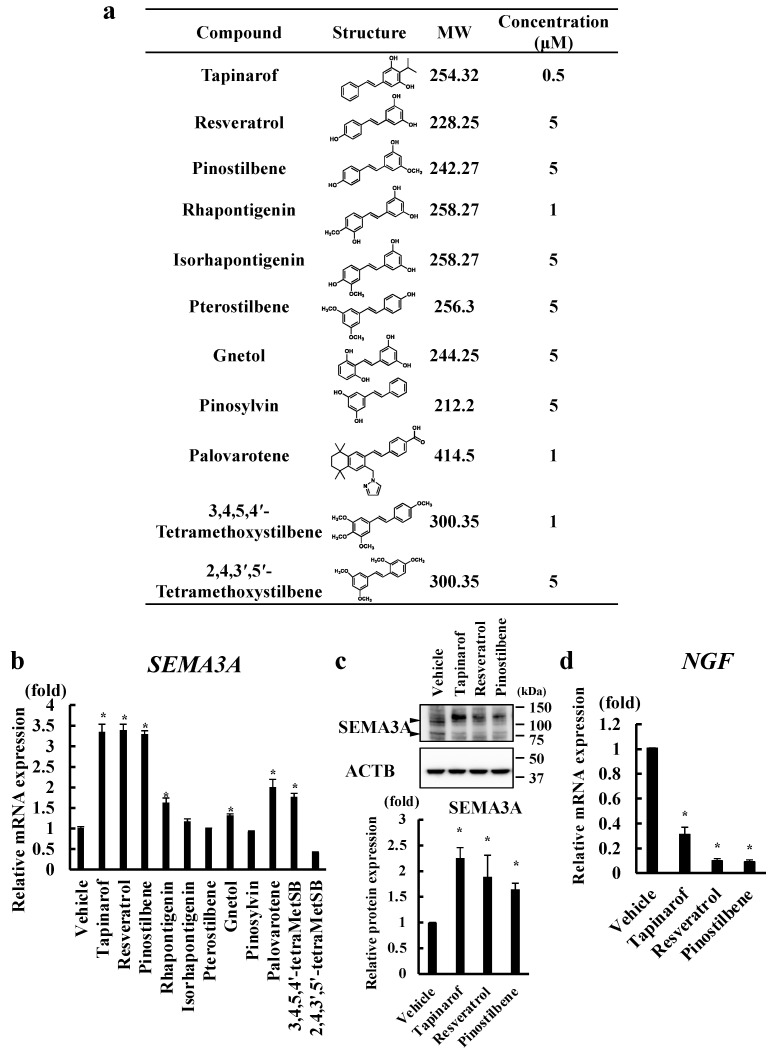
Tapinarof, resveratrol, and pinostilbene upregulated SEMA3A in NHEKs. (**a**) Chemical structures and concentrations of the tested stilbenes. (**b**–**d**) NHEKs were treated with the indicated compounds for 72 h. mRNA (**b**) and protein (**c**) levels of SEMA3A and the NGF mRNA level (**d**) were analyzed. (**b**) MetSB, methoxystilbene. (**b**,**d**) qRT-PCR. Data are expressed as mean ± S.D.; *n* = 3/group. * Significant difference between the indicated compound-treated group and the vehicle group (*p* < 0.05). (**c**) Western blotting. Data are representative of triplicate experiments with similar results. SEMA3A protein levels were normalized for ACTB protein levels using ImageJ 1.48 V. Data are expressed as mean ± S.D. * Significant difference between the indicated compound-treated group and the vehicle group (*p* < 0.05).

We selected stilbenes with a chemical structure similar to that of tapinarof, which can be purchased commercially. To examine the effects of these stilbenes on cell viability, we evaluated their cytotoxicity on NHEKs. We treated NHEKs with different concentrations of the indicated stilbenes for 24 h (Supplementary Figure S1). The highest concentration that showed no effect on the cell viability was used for the later experiments (Figure 1a). Since our previous study showed that tapinarof treatment for 72 h upregulates SEMA3A in NHEKs [20], we analyzed SEMA3A mRNA levels in NHEKs treated with the indicated stilbenes for 72 h (Figure 1b). We found that resveratrol and pinostilbene increased SEMA3A mRNA (Figure 1b) and protein (Figure 1c) levels to the same extent as tapinarof. Nerve growth factor (NGF), a neurotrophic factor, promotes the growth of nerve cells and supports the development of neural connections and neurons to reach their target tissues. Since we previously reported that tapinarof treatment inhibits NGF expression in NHEKs [20], we next analyzed NGF mRNA expression in resveratrol- and pinostilbene-treated NHEKs and found that these treatments decreased NGF mRNA levels (Figure 1d). These findings imply that resveratrol and pinostilbene may inhibit epidermal innervation by increasing SEMA3A and decreasing NGF in human keratinocytes.

### 3.2. Resveratrol and Pinostilbene Activated the AHR-NRF2 Axis in NHEKs

**Figure 2:**
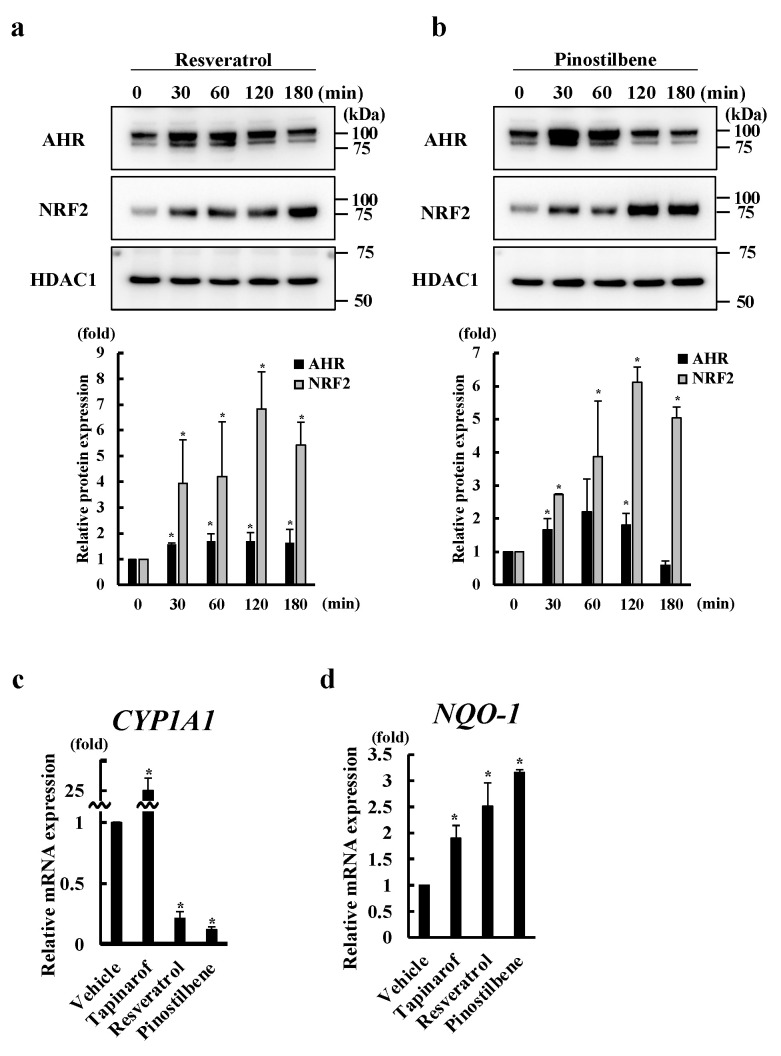

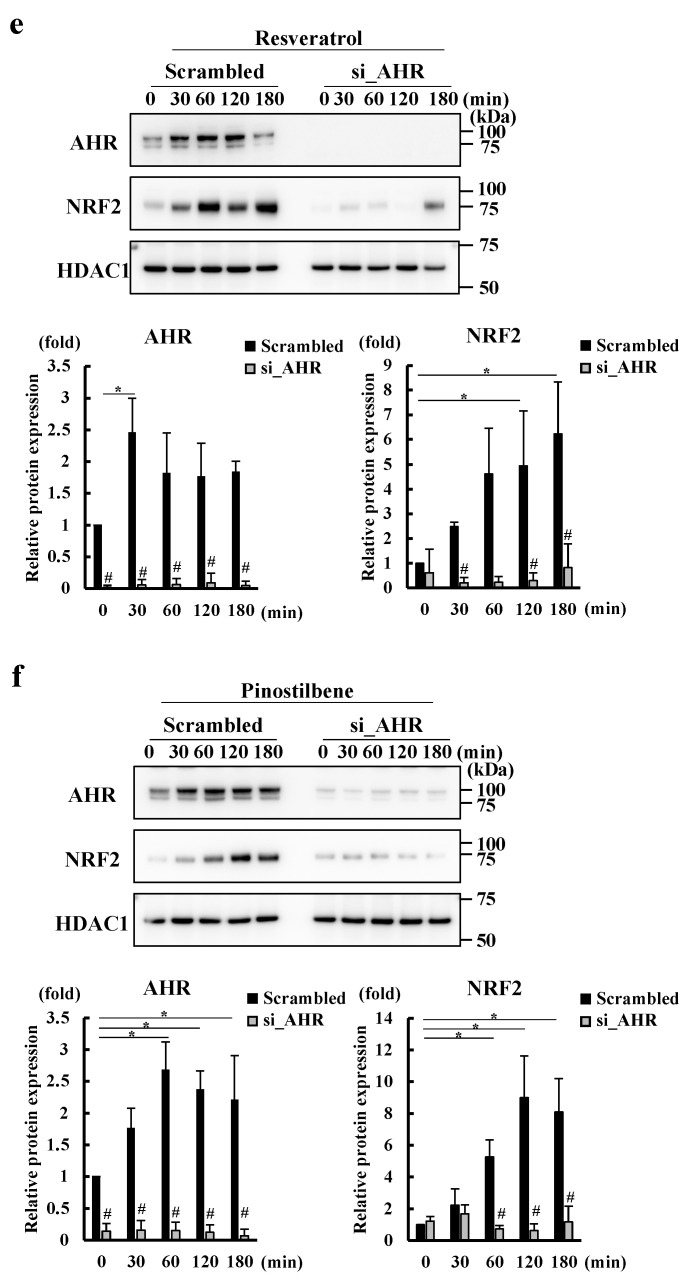
Resveratrol and pinostilbene activated the AHR-NRF2 axis in NHEKs. (**a**,**b**) Western blotting. NHEKs were treated with vehicle, resveratrol, or pinostilbene for 30, 60, 120, and 180 min and then nuclear AHR and NRF2 protein levels were analyzed. Representative images from three independent experiments are shown. Protein levels of AHR and NRF2 were normalized for HDAC1 protein levels using ImageJ. Data are expressed as mean ± S.D. * Significant difference between the indicated time administration group and the pre-administration group (*p* < 0.05). (**c**,**d**) qRT-PCR. NHEKs were treated with vehicle, resveratrol, and pinostilbene for 72 h. Data are expressed as mean ± S.D.; *n* = 3/group. * *p* < 0.05. # Significant difference between the indicated compound-treated group and the vehicle group (*p* < 0.05). (**e**,**f**) Western blotting. Scrambled or AHR siRNA-transfected NHEKs were treated with vehicle, resveratrol, and pinostilbene for 30, 60, 120, and 180 min and then nuclear AHR and NRF2 protein levels were analyzed. Representative images from three independent experiments are shown. Protein levels of AHR and NRF2 were normalized for HDAC1 protein levels using ImageJ. Data are expressed as mean ± S.D. * *p* < 0.05. # Significant difference between si_AHR-transfected group and scrambled siRNA-transfected group (*p* < 0.05).

Since we previously reported that activation of the AHR-NRF2 axis mediates SEMA3A expression in human keratinocytes [20], we further examined whether resveratrol and pinostilbene affect the activity of this axis. Although resveratrol and pinostilbene have been shown to activate AHR, which induces the nuclear translocation of AHR [29,31], this has not been tested in human keratinocytes. Furthermore, although resveratrol has been reported to activate NRF2 in human keratinocytes [32], whether pinostilbene affects NRF2 activation has not been reported. We treated NHEKs with resveratrol or pinostilbene for 30, 60, 120, and 180 min and analyzed the nuclear expression of AHR and NRF2. We found that resveratrol and pinostilbene increased the nuclear expression of AHR and NRF2 at 30 min (Figure 2a,b). We next examined whether resveratrol and pinostilbene affected AHR signal activity by measuring mRNA levels of CYP1A1, a representative AHR-mediated gene [23]. Consistent with previous reports [33,34,35], while tapinarof induced CYP1A1 expression, resveratrol and pinostilbene inhibited it (Figure 2c). We next examined whether resveratrol and pinostilbene affected NRF2 signal activity by measuring mRNA levels of NQO1, a representative NRF2-mediated gene [36]. Tapinarof, resveratrol, and pinostilbene increased mRNA levels of NQO1 (Figure 2d), indicating that they activate NRF2 in human keratinocytes. Next, we treated NHEKs in which AHR had been knocked down by siRNA transfection with resveratrol and pinostilbene for 30, 60, 120, and 180 min. AHR knockdown abolished resveratrol- and pinostilbene-induced increases in NRF2 nuclear expression (Figure 2e,f), indicating that these treatments activate NRF2 via AHR. The efficiency of the knockdown of AHR by siRNA transfection was confirmed, as shown in Figure 2e,f. These findings suggest that resveratrol and pinostilbene activate the AHR-NRF2 axis in NHEKs.

### 3.3. Resveratrol and Pinostilbene Upregulated SEMA3A via the AHR-NRF2 Axis in NHEKs

**Figure 3:**
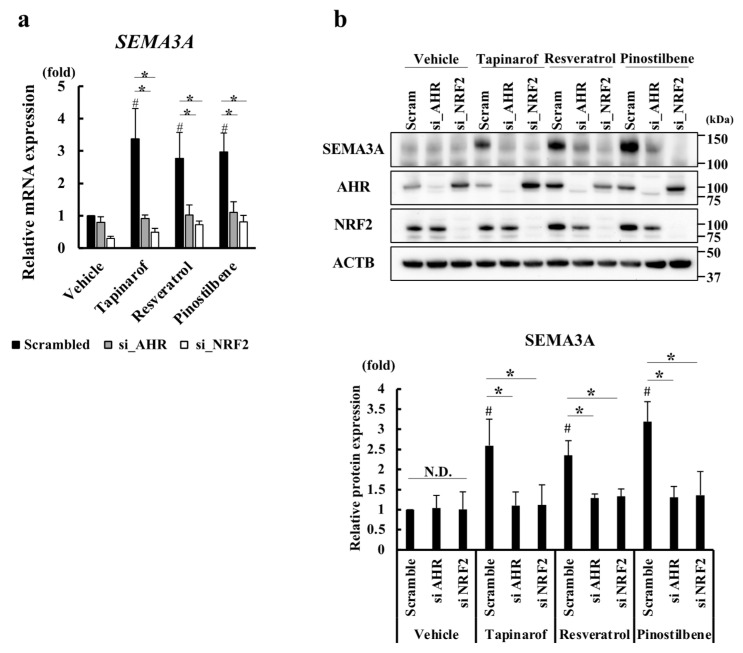
Resveratrol and pinostilbene upregulated SEMA3A via the AHR-NRF2 axis in NHEKs. (**a,b**) NHEKs were transfected with siRNA targeting AHR (si_AHR) or NRF2 (si_NRF2) or scrambled control siRNA (Scram) and then treated with vehicle, tapinarof, resveratrol, or pinostilbene for 72 h. (**a**) qRT-PCR. SEMA3A mRNA levels. Data are expressed as mean ± S.D.; *n* = 3/group. * *p* < 0.05. # Significant difference between the indicated compound-treated group and the vehicle group (*p* < 0.05). (**b**) Western blotting. SEMA3A protein levels. Representative images of three independent experiments are shown. SEMA3A protein levels were normalized for ACTB protein levels using ImageJ. Data are expressed as mean ± S.D. * *p* < 0.05. # Significant difference between the indicated compound-treated group and the vehicle group (*p* < 0.05). N.D.: No significant difference.

To further examine whether the induction of SEMA3A expression by resveratrol and pinostilbene is mediated by the AHR-NRF2 axis, we treated AHR- and NRF2- knockdown NHEKs with tapinarof, resveratrol, or pinostilbene for 72 h. Tapinarof was used as a positive control. Knockdown of AHR and NRF2 decreased the mRNA (Figure 3a) and protein levels (Figure 3b) of SEMA3A in NHEKs treated with tapinarof, resveratrol, and pinostilbene. The efficiency of the knockdown of AHR and NRF2 by siRNA transfection was confirmed, as shown in Figure 3b. These results suggest that resveratrol and pinostilbene upregulated SEMA3A via the AHR-NRF2 axis in NHEKs.

### 3.4. Resveratrol and Pinostilbene Increased the Promoter Activity of SEMA3A via NRF2 in NHEKs

**Figure 4:**
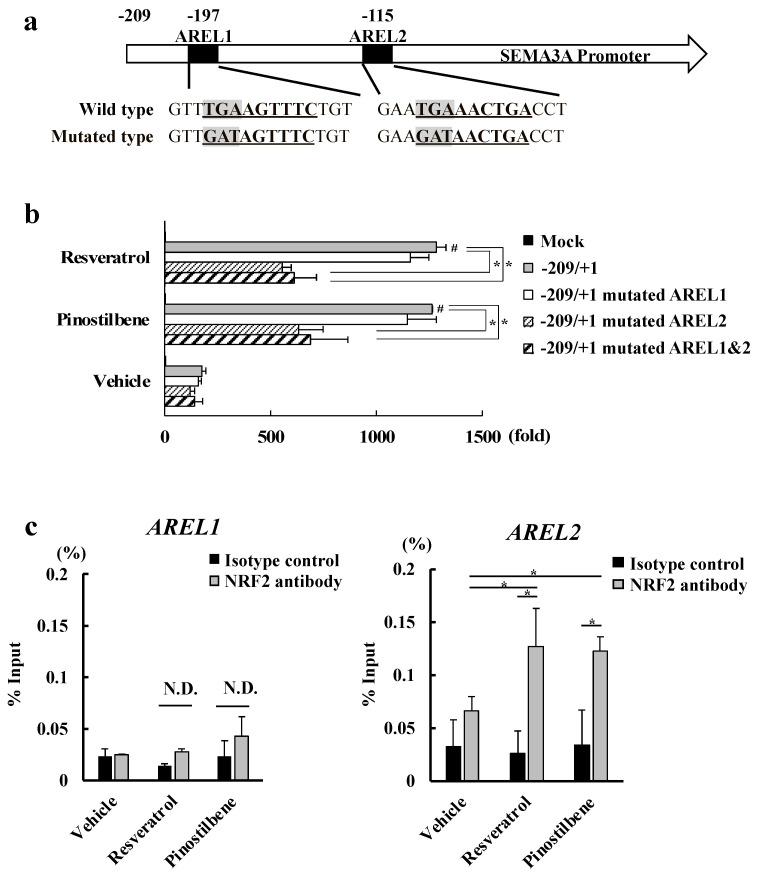
Resveratrol and pinostilbene increased the promoter activity of SEMA3A via NRF2 in NHEKs. (**a**) Locations of AREL1 and AREL2 on the SEMA3A promoter and sequences of wild-type and mutated-type AREL1 and AREL2 are indicated. (**b**) Luciferase assay. NHEKs were transfected with plasmids containing the sequence of −209/+1 SEMA3A with wild-type or mutated-type AREL1 and AREL2. After transfection, cells were stimulated with resveratrol or pinostilbene for 48 h and the relative promoter activity was determined. Data are expressed as mean ± S.D.; *n* = 3/group. * *p* < 0.05. # Significant difference between the indicated compound-treated group and the vehicle group (*p* < 0.05). (**c**) ChIP-qPCR analysis using anti-NRF2 antibody. qPCR of AREL1 and AREL2 in SEMA3A promoter was performed. Data are shown as % expression compared with that of the input samples. Data are expressed as mean ± S.D.; *n* = 3/group. * *p* < 0.05. N.D.: No significant difference.

Tapinarof enhances the activity of SEMA3A promoter by inducing NRF2 binding to antioxidant response element-like locus (AREL)2 located in the region approximately −115 bp proximal of the SEMA3A promoter in NHEKs [20]. To test whether resveratrol and pinostilbene activate this region in the same way as tapinarof, we performed a promoter assay using luciferase vectors that carry wild-type ARELs (AREL1: TGAAGTTTC and AREL2: TGAAACTGA) and mutated ARELs (mutated AREL1: GATAGTTTC and mutated AREL2: GATAACTGA) (Figure 4a). We analyzed the luciferase induction of resveratrol- and pinostilbene-treated NHEKs transfected with the constructs. The increases in luciferase induction by resveratrol and pinostilbene were reduced in NHEKs transfected with the construct containing mutated AREL2 (Figure 4b), suggesting that NRF2 binds to AREL2. Finally, we performed ChIP-qPCR analysis to determine the binding of NRF2 to AREL2. We extracted chromatin from NHEKs treated with resveratrol and pinostilbene for 48 h and then reacted the chromatin with anti-NRF2 antibody or isotype control IgG. Subsequently, we precipitated DNAs with protein A agarose and subjected them to qPCR. We confirmed that NRF2 activated by resveratrol and pinostilbene bound to AREL2, not AREL1 (Figure 4c). These results show that resveratrol and pinostilbene increased the promoter activity of SEMA3A via NRF2 in NHEKs.

## 4. Discussion

Resveratrol is a natural polyphenol found in berries and grapes that has been reported to exert cytoprotective effects through antioxidant activity in human keratinocytes [37,38,39]. Several studies have reported the therapeutic effect of resveratrol on AD in mouse models. Oral administration of resveratrol inhibited the development of skin lesions in AD induced by the topical application of mite antigen and 2,4-dinitrochlorobenzene (DNCB) [40,41]. In addition, resveratrol-enriched rice intake has been shown to inhibit pruritus and subsequently reduce the frequency of scratching in a DNCB-induced AD mouse model [42]. There are a few reports of the treatment of AD by the topical application of resveratrol. Recently, the topical application of resveratrol-loaded nanoemulgel or piceatannol, a metabolite of resveratrol, has been reported to attenuate the development of dermatitis in an AD model mouse [43] (Nene S). While the therapeutic effect of resveratrol on AD has been shown, the mechanism by which it alleviates AD is still unclear. It has been reported that resveratrol suppresses the activation of NF-κBp65 [44], Janus kinase (JAK) 1 [45], signal transducer and activator of transcription 3 (Stat3) [46], and sphingosine kinase 1 [46], resulting in the downregulation of inflammatory cytokines. However, the possibility that resveratrol acts on AHR and NRF2 to improve the pathogenesis of AD, as shown by this study, has not been reported.

Pinostilbene, a naturally occurring methylated derivative of resveratrol, has been reported to exert anticancer activity against human colon cancer cells and human oral squamous cell carcinoma cells [47,48]. Pinostilbene has also been reported to exert anti-inflammatory effects by inhibiting COX-1 and COX-2 [49] and cytoprotective activity against a neurotoxin [50]. However, few studies have focused on the therapeutic effects of pinostilbene on skin diseases. One report has shown that pinostilbene suppresses melanin production in human melanocytes, which suggests that it could be used in functional cosmetics for the treatment and prevention of pigmentation disorders such as melasma [51]. Meanwhile, the pharmacological effects of pinostilbene on human keratinocytes have yet to be verified. The present study has shown that resveratrol and pinostilbene activate the AHR-NRF2 axis in human keratinocytes, which may allow for a more precise investigation of their effects on inflammatory skin diseases. 

We have shown that AHR is required for the activation of NRF2 induced by resveratrol and pinostilbene (Figure 2e,f). Therefore, we believe that the binding of resveratrol and pinostilbene to the AHR activates NRF2 in human keratinocytes; however, there is a possibility that another mechanism is involved. Resveratrol has been reported to bind to the binding site of HDACs and interferes with their function [52]. It has also been reported that HDAC inhibitors increase the recruitment of AHR to target gene promoters [53], which suggests that resveratrol may activate AHR signaling by inhibiting HDAC activity. Furthermore, resveratrol has been reported to increase NRF2 expression and induce methylation of the NRF2 promoter, thereby regulating the expression of NRF2 target genes [54]. Therefore, epigenetic modulation by resveratrol may affect the activation of the AHR–NRF2 axis. To the best of our knowledge, there are no reports of the effects of pinostilbene on the activity of HDACs.

Resveratrol is environmentally unstable, easily deformable, and highly susceptible to ultraviolet light, air, and high pH [55]; these features seriously obstruct its bioavailability. Therefore, many resveratrol derivatives have been investigated, and studies have found that methylated resveratrol derivatives have higher bioavailability [35]. However, pharmacokinetic studies in which resveratrol or pinostilbene was administered orally to rats have shown that their bioavailability is low [56]. It is thus thought that the oral administration of resveratrol and pinostilbene will be difficult to use for treating AD.

In the field of dermatology, compounds with low bioavailability can be applied directly to lesions using a topical agent. The stratum corneum, the outermost layer of the skin, is predominantly hydrophobic, whereas the epidermis is predominantly aqueous. Therefore, the ideal topical drug should be of low molecular weight (<500 Da) [57] and have both hydrophobic and hydrophilic properties to cross the stratum corneum and the aqueous epidermis [58]. The molecular size of resveratrol is 228.25 and that of pinostilbene is 242.27 (Figure 1a). Resveratrol and pinostilbene have both hydrophobic and hydrophilic properties. Indeed, the penetration of topically applied resveratrol both in vitro and in vivo has been evaluated, with the results showing that topically applied resveratrol can maintain its antioxidant efficacy after penetration [59]. However, no studies have been conducted on pinostilbene.

While tapinarof, resveratrol, and pinostilbene share the capacity to induce SEMA3A expression via the AHR-NRF2 axis in NHEKs, tapinarof induces CYP1A1 expression, whereas resveratrol and pinostilbene inhibit it. Polyphenols have been suggested to act as cell-specific agonists or antagonists of AHR [60]. In human keratinocytes, phytochemicals that exert their antioxidant effects via AHR and NRF2 have been classified into three groups according to their ability to increase or decrease AHR and CYP1A1 activity. Group 1 includes AHR agonists with NRF2 agonist activity, such as *Opuntia ficus-indica* extract, *Houttuynia cordata* extract, *Bidens pilosa* extract, and cynaropicrin. Group 2 includes AHR antagonists with NRF2 agonist activity, such as cinnamaldehyde and epigallocatechin gallate. Group 3 includes CYP1A1 inhibitors with NRF2 agonist activity, such as quercetin, kaempferol, pterostilbene, and resveratrol [61]. Pinostilbene has also been reported to inhibit CYP1A1 activity [35]. The mechanism by which resveratrol induces the nuclear translocation of AHR in human epidermal cells despite CYP1A1 inhibition is thought to involve CYP1A1 inhibition, reducing the clearance of endogenous AHR agonists such as 6-formylindolo[3,2-b]carbazole (FICZ), which in turn activates AHR. FICZ is produced by the ultraviolet-induced conformational change in tryptophan, an essential amino acid [62]. Indeed, when resveratrol was administered to human keratinocytes under conditions with a medium lacking tryptophan to prevent the formation of FICZ, activation of AHR was not observed [63]. These results suggest that resveratrol and pinostilbene may prolong the presence of endogenous AHR agonist by inhibiting CYP1A1, thereby activating the AHR-NRF2 axis and increasing SEMA3A expression. It is thus possible that, when resveratrol and pinostilbene are applied topically in combination with tapinarof, the metabolic degradation of tapinarof may be delayed, leading to enhanced activation of the AHR-NRF2 axis, which may increase the pharmacological effect of tapinarof. In addition, clinical trials of tapinarof for AD have shown a high safety profile, but folliculitis and contact dermatitis at the site of application have been noted as adverse events [64,65]. Therefore, there is concern that these adverse events may increase when tapinarof is combined with resveratrol or pinostilbene; however, further studies including some performed in vivo are needed.

As mentioned above, the topical application of resveratrol and pinostilbene may be beneficial for the treatment of AD and dry skin, but the fact that they could then be exposed to UV radiation should be considered. Resveratrol has been reported to increase UV-induced DNA damage in human keratinocytes and the production of inflammatory cytokines such as IL-8. It has been postulated that the mechanism behind this involves resveratrol inhibiting CYP1A1, thereby delaying the degradation of UV-induced FICZ [62]. FICZ is reported to act as a nanomolar photosensitizer that enhances UVA-induced oxidative stress, which is independent of AHR ligand activity [63]. It has also been reported that SEMA3A expression is increased, accompanied by decreased nerve fibers in diabetic small fiber neuropathy, resulting in sensory impairment [66]. Therefore, topical application of resveratrol and pinostilbene to diabetic patients should be performed with caution to avoid exacerbating sensory disturbances. It has also been reported that Sema3A expression is increased in mice induced to develop a nickel allergy [67]. Specific deletion of Sema3A in keratinocytes in these mice reduced nickel allergy-induced ear swelling. These results suggest that Sema3A is involved in the development of nickel allergy. Although Sema3A induced T-cell differentiation into the Th1 subset and alleviated Th2 responses during nickel allergy [67], which contributes to reducing AD symptoms, some AD patients have comorbid nickel allergy [68], so physicians need to inquire about metal allergies.

In conclusion, we have shown that resveratrol and pinostilbene, in addition to tapinarof, induce SEMA3A expression via the AHR–NRF2 axis in human keratinocytes. Since SEMA 3A prevents epidermal innervation, these results may be useful for the development of new topical treatments for AD and dry skin using antioxidants.

## Data Availability

The authors confirm that the data supporting the findings of this study are available within this article and the Supplementary Materials.

## Supplementary Materials

The following supporting information can be downloaded at: https://www.mdpi.com/article/10.3390/antiox13060732/s1. Supplementary Figure S1: Effect of stilbenes on cell viability in NHEKs; Supplementary Table S1: List of primers and probe.

## Abbreviations

CCL: chemokine C-C motif ligand; CXCL, C-X-C motif chemokine ligand; JAK, Janus kinase; MAPK, mitogen-activated protein kinase.
