# Supplementary material for: Induction of Semaphorin 3A by Resveratrol and Pinostilbene via Activation of the AHR-NRF2 Axis in Human Keratinocytes
Source: Antioxidants (Basel). 2024 Jun 17;13(6):732. doi: 10.3390/antiox13060732 (PMC11201291; doi:10.3390/antiox13060732)
Supplement: Supplementary file 1 [file antioxidants-13-00732-s001.zip › antioxidants-3032337-supplementary.pdf]

Supplementary Materials:

Supplementary Figure S1. Effect of stilbenes on cell viability in NHEKs.

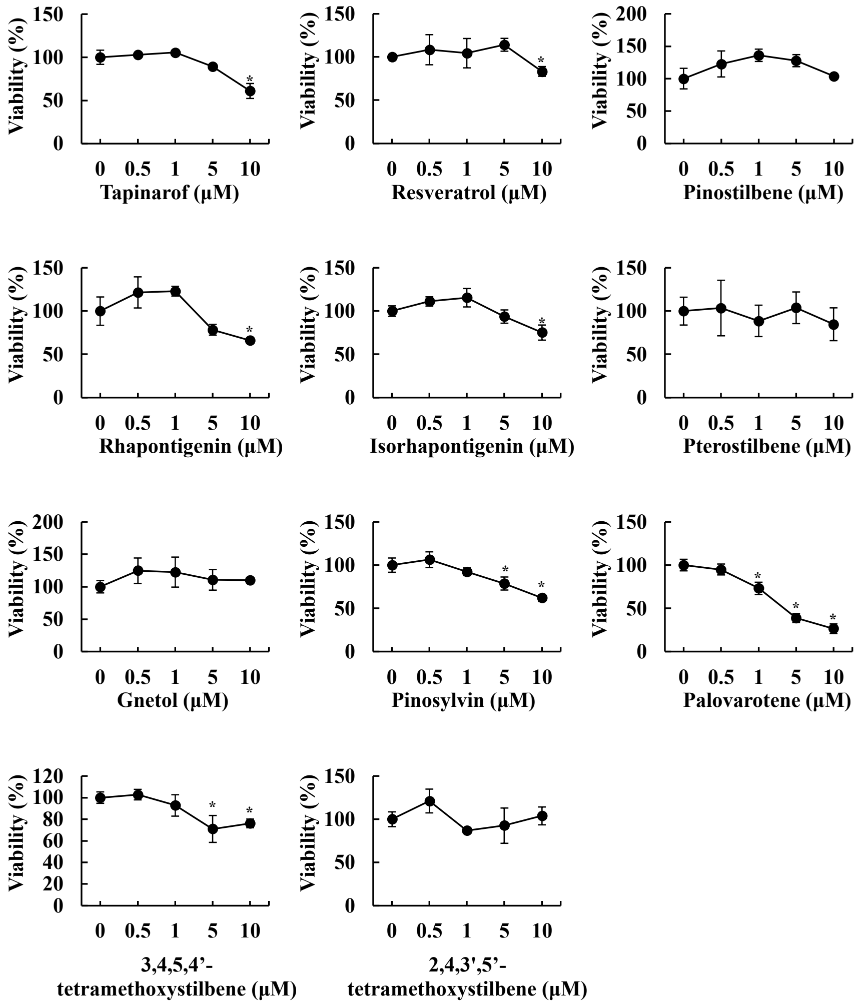

NHEKs were treated with the indicated stilbenes for 24 h. Cell viability was measured using Cell Counting Kit-8 (Dojindo, Tokyo, Japan). The results are presented as the absorbance relative to that of vehicle-treated NHEKs. Data are expressed as mean ± S.D.; N = 3/group. *Significant difference between indicated compound-treated group and vehicle group ($p < 0.05$).

Supplementary Table S1. List of primers and probe

Gene Symbol	GenBank accession No.	Forward Primer 5'-3'	Reverse Primer 5'-3'
YWHAZ	NM_145690	tgaggttgccgctggtgatg	cagtctgataggatgtgttggttgc
NGF	NM_002506	catgctggacccaagctca	cctgcagggacattgctctc
CYP1A1	NM_001319217	tagacactgatctggctgcag	gggaaggctccatcagcatc
NQO-1	NM_000903	gaagagcactgatcgtactggc	ggatactgaaagttcgcaggg
AHR	NM_001621	caaatccttccaagcggcata	cgctgagcctaagaactgaaag
NRF2	NM_006164	tcagcgacggaaagagtatga	ccactggtttctgactggatgt
*SEMA3A*_AREL1		cctcattatccggtgcctg	tcaagacctcatggcaacact
*SEMA3A*_AREL2		agtgttgccatgaggtcttga	ctgtattgtgcggccagaga

Gene Symbol	GenBank accession No.	Taqman probe
YWHAZ	NM_145690	Hs01122445_g1
SEMA3A	NM_006080	Hs00173810_m1
